# Supplementary material for: Air Quality during COVID-19 in Four Megacities: Lessons and Challenges for Public Health
Source: Int J Environ Res Public Health. 2020 Jul 14;17(14):5067. doi: 10.3390/ijerph17145067 (PMC7400368; doi:10.3390/ijerph17145067)
Supplement: Supplementary file 1 [file ijerph-17-05067-s001.docx]

Supplementary Material

S1. Descriptions of Containment Measures Implemented

Containment measures in São Paulo Metropolitan Area:

On February 26, 2020, the first imported case was confirmed in the city of São Paulo. However, in a few days, cases started to pop in Rio de Janeiro and then in other capitals, and also to increase in São Paulo, indicating communal transmission (‘community spread’). In an attempt to gain time for the preparation of health services and to avoid a very steep curve in the number of cases, the city of São Paulo, being the largest and having the majority of cases, declared it mandatory that students from all levels not go to school, using a gradual scale starting on March 13. On March 23 all the schools and universities were closed. Distance learning approaches were applied on a large scale.

From March 21, all commercial, artistic and public buildings were required to close their doors to the public (this was later extended to May 11). Only pharmacies, grocery stores and bakeries were allowed to stay open. Restaurants were only allowed to serve food through delivery services. Additionally, all workers from public and/or private institutions were required to stay home and, when possible, to work remotely using phone calls or the Internet. Parks, museums, libraries and cultural centers were also closed. Many cities in the metropolitan area adopted these actions either as recommendations or as requirements. However, this was not enough to postpone the rising trend of infected cases by the novel Coronavirus.

From March 23, the Rodízio (car restriction according to license plate) was suspended by local authorities in downtown São Paulo city, parks were closed, and people were strongly asked to stay home. The closing of shops, offices, and restaurants became mandatory and parties, religious ceremonies and gatherings of large groups of people were prohibited in the city. A flu vaccination campaign was started for elderlies and health workers. Many Primary Care Health Centers observed long lines of cars because people could receive vaccines inside the cars.

The circulation of commercial trucks was less affected because of the need for food and medical device distribution around the city and in the country as a whole. Heavy motorcycle circulation continued and probably increased within the city because of increased E-commerce and food delivery demands.

Containment measures in New York City:

After both travel related cases and community contact transmission of COVID-19 had been documented, on March 7, 2020, there was a Declaration of State Disaster Emergency for the entire State of New York (State of New York, 2020). On March 12, 2020, to be effective on March 13, Executive Order 202 was enacted, to be in effect without any suspension or modification until April 11, 2020. This order included:

Suspension of Law allowing residents of Nursing Homes to vote, and Guidance on visitation at Nursing Homes to prevent and control COVID-19. Canceling of any large gatherings or events for which attendance was expected to be in excess of 500 people. Operation at no greater than 50% occupancy or 50% of seating capacity for business or public accommodations and events with fewer than 500 people.

These two directives did not apply to schools, hospitals, nursing homes, medical offices, mass transit or mass transit facilities, government facilities, law enforcement facilities, or retail establishments including grocery stores. On March 22, 2020, the New York on Pause Executive Order declared that: 100% of the workforce must stay home, excluding essential services; all non-essential gatherings of individuals of any size for any reason were temporarily banned; statewide school closures were extended until April 15.

NY City started piloting closing streets to vehicles and opening them to pedestrians as part of the city plans to address the lack of adherence to social distancing protocols. All non-essential businesses statewide must close in-office personnel functions. Bars and Restaurants are closed but take out could be ordered. Residents were advised stay in their houses after 8 p.m. Essential services that could stay open were specified: Grocery stores and health care facilities.

Matilda Law was passed to protect the most vulnerable (those +70 y, with underlying illness and with compromised immune systems), who were required to stay home and limit home visitation to immediate family.

There was a self-quarantining guidance for NY residents who were leaving the area. However, it did not apply to delivery workers, truck drivers and other people driving into the city to deliver necessary supplies. It was recommended for all people to stay home when sick.

Containment measures in Los Angeles:

On March 16, 2020, restrictions on public events began. On March 19, 2020 the quarantine was declared via the “Safer at Home” emergency order. On March 25, 2020, expecting a wave of coronavirus cases to wash over Los Angeles, health officials announced more stringent isolation and quarantine orders. Given the rapid spread of the disease, anyone confirmed to have the disease or believed to have it by their physician, had to remain in isolation, as well as those close to them. Residents of LA county and across the state were under orders to remain at home as much as possible and engage in social distancing when they were outside the home.

The restrictions were ramped up over the weekend in response to continued large-scale gatherings of people at beaches -- most notably the Venice boardwalk -- and on hiking trails. Saturday's enhanced order, of March 28, also clarified that golf courses and personal grooming services -- including hair and nail salons -- are nonessential services and should remain closed. Businesses considered essential and permitted to remain open included hardware stores, repair shops, media outlets, banks, laundromats, dry-cleaners and pet supply stores.

In Los Angeles County: all Roman Catholic churches in the Archdiocese of Los Angeles were to be closed; the Malibu Pier, considered a state park, including its shops and restaurants, was closed to the public starting March 25 to prevent crowding.

Containment measures in Paris

The first cases of coronavirus in France were confirmed on January 24, 2020, but more severe measures were implemented only in March (including the safety and maintenance of the municipal elections), such as restrictions of public gathering to 1,000 people, closing of schools on March 12, and economic assistance measures. On March 14, markets, restaurants, coffee-shops and all non-necessary commerce were closed.

On March 17, mandatory confinement measures were implemented in France. The measures were announced after the French President Macron’s public television speech on March 16 that explained the motivations and seriousness of intervention. Borders with non-European countries were closed. These measures limited work related mobility to professional activities that could not be replaced by teleworking. Trips related to the purchase of goods for professional activities were authorized as well as those for basic/ necessary food shopping; money withdrawal was possible in authorized establishments. Health consultations or care were allowed in very precise circumstances or for patients with long-term infections. On March 23, borders within the European Union were also closed (France, 2020).

Other authorized trips included those related to family situations such as childcare or assistance for vulnerable populations. All trips were limited to 1 hour per day within a one-kilometer radius from home and had to be linked to individual physical activity, walks with some home family members and for domestic animals. Another particularity of the confinement rules in France was the need to carry an authorization justifying trips. All schools were closed.

References

France, 2020. Décret n° 2020-293 du 23 mars 2020 prescrivant les mesures générales nécessaires pour faire face à l’épidémie de covid-19 dans le cadre de l’état d’urgence sanitaire.

State of New York, 2020. Executive Order Number 202.

**Table S1.** Scenario of air quality in São Paulo for classical pollutants, 2010 - 2019.

| **Pollutant** | **Period** | **Number of monitoring stations** | **Maximum annual average concentration among monitoring stations** | **Highest short-term concentration ^(b)^** | **Number of times above WHO’s guidelines** | | |
| --- | --- | --- | --- | --- | --- | --- | --- |
|  |  |  |  |  | **Total** | **Average per year** | **Average per year per station** |
| Carbon Monoxide  (ppm) | 10 years^(a)^ | 16 | 1.2 | 8.5 | 0 | 0 | 0.0 |
|  | 2019 | 15 | 1 | 5.7 | 0 | 0 | 0.0 |
| Nitrogen Dioxide  (μg/m^3^) | 10 years | 17 | 5 | 350.0 | 125 | 13 | 0.7 |
|  | 2019 | 13 | 62 | 226.0 | 4 | 4 | 0.3 |
| Sulfur Dioxide  (μg/m^3^) | 10 years | 6 | 5 | 31.0 | 5 | 0.4 | 0.1 |
|  | 2019 | 8 | 3 | 21.0 | 1 | 1 | 0.13 |
| PM_10_  (μg/m^3^) | 10 years | 21 | 43 | 174.0 | 8.412 | 841 | 40.1 |
|  | 2019 | 21 | 43 | 119.0 | 642 | 642 | 30.6 |
| PM_2.5_  (μg/m^3^) | 10 years | 6 | 22 | 89.0 | 2.738 | 274 | 45.6 |
|  | 2019 | 16 | 21 | 83.0 | 762 | 762 | 47.6 |
| Ozone  (μg/m^3^) | 10 years | 20 | 44 | 234.0 | 8.516 | 852 | 42.6 |
|  | 2019 | 22 | 58 | 208.0 | 1.040 | 1.040 | 47.3 |

^a^ from 2010 to 2019

^b^ short-term concentration means 8 hours for carbon monoxide and ozone, 1 hour for nitrogen dioxide and 24 hours for sulfur dioxide and particulate matter (PM_10_ and PM_2.5_).

Source of data: QUALAR


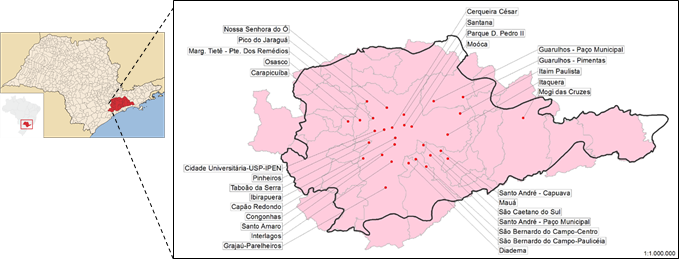


**Figure S1.**  Brazil highlighting São Paulo State, São Paulo Metropolitan Area and CETESB Air Quality Stations Source: CETESB, 2019; Image:SaoPauloMesoMicroMunicip.svg.


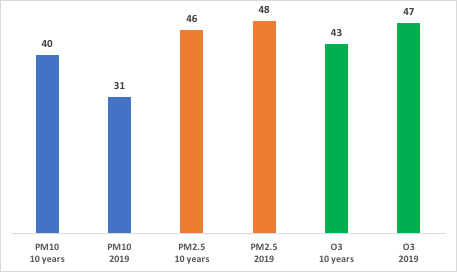


**Figure S2.** Average number of times, per year and per monitoring station, that concentrations of PM_10_, PM_2.5_ and ozone were above WHO's guidelines in 10 years (2010-2019) and in 2019.


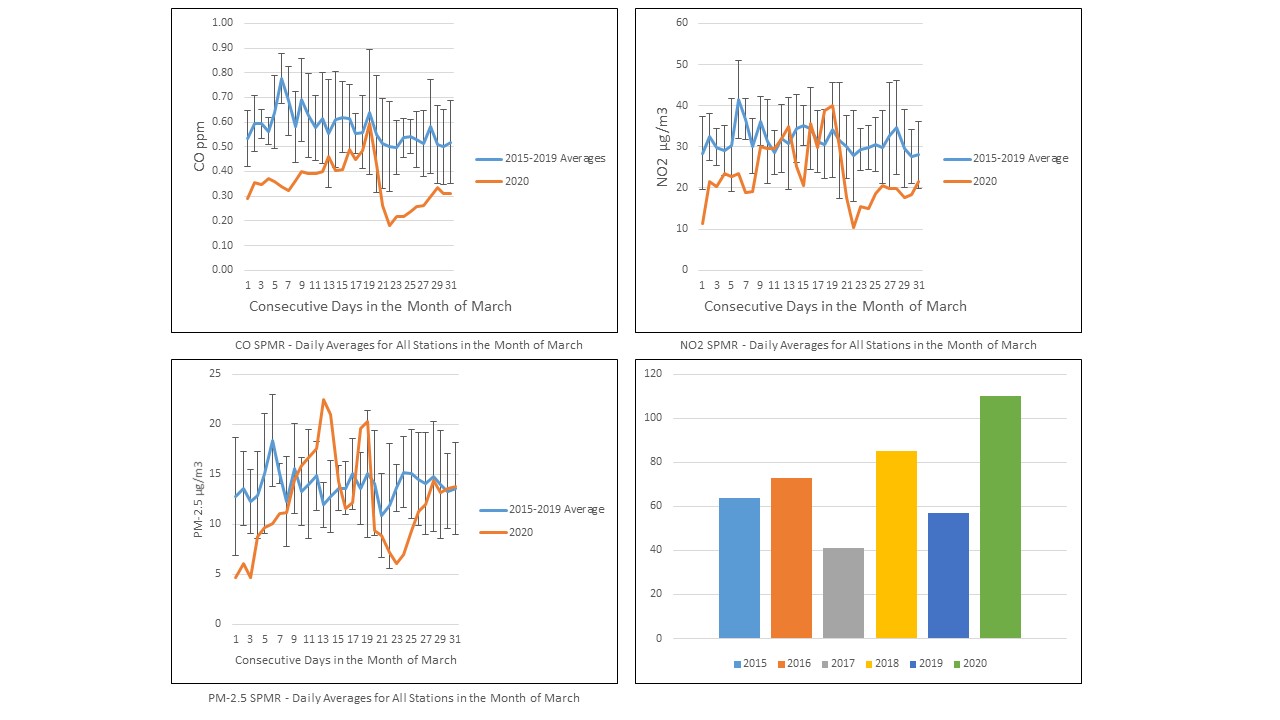


**Figure S3.** Ozone – number of exceedances of the WHO Guideline value (100 µg/m^3^) in the SPMA.

|  |  |
| --- | --- |
| (**a**) | (**b**) |

**Figure S4.** São Paulo city daily average for temperature, relative humidity, wind speed, wind direction and precipitation in the month of March 2020.
